# Prediction of Adsorption and Diffusion Behaviors of CO_2_ and CH_4_ in All-Silica Zeolites Using Molecular Simulation

**DOI:** 10.3390/membranes11060392

**Published:** 2021-05-26

**Authors:** Yasuhisa Hasegawa, Chie Abe

**Affiliations:** National Institute of Advanced Industrial Science and Technology, Research Institute of Chemical Process Technology, 4-2-1, Nigatake, Sendai 983-8551, Japan; abe-chie@aist.go.jp

**Keywords:** grand canonical Monte Carlo, adsorption isotherm, all-silica zeolites, molecular dynamic, self-diffusivity

## Abstract

Computational chemistry is a promising technique for the prediction of material properties. Adsorption and diffusion behaviors in zeolite micropores are important for zeolite membranes. In this study, we investigated novel non-bonding interaction parameters of all-silica zeolites for the prediction of the adsorption and diffusion behaviors by focusing on the Si atom of zeolite frameworks. Our parameters (σ = 0.421 nm, ε = 0.954 kJ mol^−1^, and q = +1.10 e) were close to theoretically derived values, and the adsorption isotherms of CO_2_ and CH_4_ on several zeolites could be predicted with high accuracy. Furthermore, the parameters gave the suitable self-diffusivities of CO_2_ and CH_4_ within MFI-type zeolite micropores through molecular dynamics simulation. Those suggest that our derived parameters are useful for selecting zeolite structure as the membrane material.

## 1. Introduction

Zeolites are aluminosilicate compounds with regular-sized micropore structures, and they show unique adsorption behaviors due to their micropore structures. Therefore, they are often used as adsorbents and catalysts. It has been possible to form polycrystalline zeolite layers on porous substrates since the 1990s [1,2,3,4,5,6,7], and zeolite membranes have been attracting much attention as an energy-saving separation technology since then. Furthermore, new zeolite structures and their adsorption and diffusion behaviors have been reported. However, it has been difficult to select suitable zeolite structures and compositions for membrane separation.

There are already many studies about the prediction of the adsorption and diffusion behaviors in zeolites using grand canonical Monte Carlo (GCMC) and molecular dynamics (MC) [8,9,10,11,12,13,14]. However, valid parameters for many zeolites have not yet been reported. Talu et al. [9] assumed that the adsorbed molecule in zeolite micropores interacts with the oxygen atoms of zeolites, and the adsorption isotherms of CO_2_ and CH_4_ on all-silica MFI-type zeolite (silicalite-1) were simulated. Although the adsorption properties for the MFI-type zeolite could be predicted, the parameters were not valid for other types of zeolites.

The parameters for adsorbates and zeolites are necessary to predict these behaviors with high accuracy. For adsorbate molecules, Sun et al. [15,16] and Siepmann et al. [17,18,19,20] investigated the suitable parameters for Lennard-Jones 9-6 and 12-6 potentials, respectively. Their parameters can describe vapor–liquid equilibria. Rahmati et al. [12] tried to predict the adsorption isotherms on all-silica zeolites using parameters reported by Sun et al. However, the simulated amounts of adsorbed CO_2_ and CH_4_ on MFI and DDR-type zeolites were several times higher than those in experimental results. Although Bai et al. [11] and Vujic et al. [14] reported on the simulation parameters of all-silica zeolites for Lennard Jones 12-6 potential, their parameters could not be applied to many zeolites.

In our study, we have found the suitable parameters for many kinds of all-silica zeolites by focusing on the silicon atom of all-silica zeolites, and the simulated adsorption isotherms were compared to experimental results to check the validation of our parameters in this paper. Furthermore, we discussed the simulated self-diffusivities in MFI-type zeolite micropores.

## 2. Theory

The interaction between adsorbate and adsorbent atoms is described as the sum of interactions between bonded and non-bonded atoms as follows:

(1) $$E_{t}=E_{\mathrm{bond}}+E_{\mathrm{non}\text{-}\mathrm{bond}}.$$

The interaction between bonded atoms is calculated as the sum of bond stretching and angle bending as follows:

(2) $$E_{\mathrm{bond}}=E_{\mathrm{bond}\text{-}\mathrm{stretch}}+E_{\mathrm{angle}\text{-}\mathrm{bend}},$$

(3) $$E_{\mathrm{bond}\text{-}\mathrm{strech}}=\frac{1}{2}k_{b}{\left(r-r_{0}\right)}^{2},$$

(4) $$E_{\mathrm{angle}\text{-}\mathrm{bend}}=\frac{1}{2}k_{\theta }{\left(\theta -\theta_{0}\right)}^{2},$$

where *k*_b_ and *k_θ_* are the force constants for bond stretching and angle bending, respectively. The interaction between the non-bonded atom pair is calculated as the sum of van der Waals and coulomb interactions as follows:

(5) $$E_{\mathrm{non}\text{-}\mathrm{bond}}=E_{\mathrm{vdW}}+E_{\mathrm{coulomb}},$$

(6) $$E_{\mathrm{vdW}}=4\varepsilon_{ij}\left[{\left(\frac{\sigma_{ij}}{r_{ij}}\right)}^{12}-{\left(\frac{\sigma_{ij}}{r_{ij}}\right)}^{6}\right],$$

(7) $$E_{\mathrm{coulomb}}=\frac{1}{4\pi \varepsilon_{0}}\cdot \frac{q_{i}q_{j}}{r_{ij}},$$

where the depth of interaction *ε_ij_* and zero-interaction distance *σ_ij_* for the pair of different atoms are calculated as follows:

(8) $$\varepsilon_{ij}=\sqrt{\varepsilon_{i}\varepsilon_{j}},$$

(9) $$\sigma_{ij}=\frac{1}{2}(\sigma_{i}+\sigma_{j}).$$

When two atoms are in the same structure and separated by three covalent bonds (so-called 1–4 interaction), the interaction is treated by non-bonded interaction with a scaling factor of 0.5. Non-bonded interaction is ignored for the directly bonded atoms (1–2 interaction) and two atoms separated by two bonds (1–3 interaction), since they are included in the bond-stretching and angle-bending interactions.

## 3. Materials and Methods

### 3.1. Synthesis of All-Silica Zeolites

#### 3.1.1. BEA-Type Zeolite

All-silica BEA-type zeolite was synthesized via a hydrothermal process [13]. Tetraethyl orthosilicate (Tokyo Chemicals Industry, Tokyo, Japan) was added to tetraethylammonium hydroxide solution (TEAOH, 40 wt.%, Aldrich, St Louis, MO, USA), and the mixture was stirred overnight at 313 K for hydrolysis. After the by-produced ethanol was vaporized at 373 K, water and hydrogen fluoride solution (46%, Wako Pure Chemicals Industry, Tokyo, Japan) was added. The mixture was mixed using a spatula to obtain a homogeneous white paste. The molar composition of the paste was 1 SiO_2_: 0.5 TEAOH: 0.5 HF: 7 H_2_O. The paste was transferred into an autoclave, and a hydrothermal reaction was carried out at 423 K for 14 days under a rotation of 60 rpm. After the reaction, solids were recovered by filtration, washed with distilled water, dried at 383 K overnight, and calcined in air at 773 K for 10 h to remove the structure-directing agent. Thus, the all-silica BEA-type zeolite particles were obtained.

#### 3.1.2. CDO-Type Zeolite

CDO-type zeolite particles were prepared via the structure conversion of layered silicate PLS-1 [21]. Potassium hydroxide, tetramethylammonium hydroxide solution (TMAOH, Aldrich), and 1,4-dioxane (Dx, Wako Pure Chemical industry) were dissolved into distilled water. Fumed silica particles (Cabot Corp., Cab-o-sil M5) were added to the solution, and the gel was transferred into the autoclave after mixing at room temperature for 6 h. The molar composition of the gel was 1 SiO_2_: 0.007K_2_O: 0.22 TMAOH: 3.4 Dx: 16 H_2_O. A hydrothermal reaction was carried out at 423 K for 14 days. Solids were recovered by centrifugal separation, washed with distilled water, and dried in air at 383 K overnight to obtain layered silicate PLS-1 particles. Then, the PLS-1 particles were ion-exchanged in a 0.1 mol/L-HCl solution at 333 K for 3 h. The particles were recovered by filtration, washed with distilled water, and dried in air at 383 K overnight. Finally, the PLS-1 particles were calcined in air at 873 K for 2 h to convert the structure from PLS-1 to CDO-type zeolite.

#### 3.1.3. CHA-Type Zeolite

All-silica CHA-type zeolite was synthesized hydrothermally [22]. Tetraethyl orthosilicate and *N,N,N*-trimethyl-1-ammonium hydroxide solution (TMAAOH, Sachem, Osaka, Japan) were mixed and stirred for 2 h at 373 K for hydrolysis. A hydrofluoric acid solution (Wako Pure Chemicals Industry) was added to the solution, after the solution was concentrated via evaporation. The molar composition of the mixture was 1SiO_2_: 0.5TMAAOH: 0.5HF: 3H_2_O. The mixture was transferred to the autoclave, and a hydrothermal reaction was carried out at 423 K for 24 h. Solids were recovered by filtration and washed with water until the pH of the washing water became neutral. After drying at 383 K overnight, the particles were calcined in air at 823 K for 10 h to burn out the structure-directing agent.

#### 3.1.4. DDR-Type Zeolite

All-silica DDR-type zeolite was synthesized via a hydrothermal reaction [23]. Adamantamine (ADA, Tokyo Chemicals Industry) was dissolved into ethylenediamine (EDA, Tokyo Chemicals Industry), and distilled water was added to the solution. The mixture was stirred for 1 h at 373 K after stirring at room temperature for 1 h, and then, the solution was cooled in an ice bath. Tetramethyl orthosilicate (Tokyo Chemicals Industry) was added to the solution, and the mixture was stirred at 373 K overnight to obtain a clear solution. The molar composition of the solution was 1SiO_2_: 4.1EDA: 0.47ADA: 112H_2_O. The solution was added to the autoclave, and a hydrothermal process was carried out at 423 K for 25 days. Solids were recovered by filtration, washed with water, and dried in air at 383 K overnight. Finally, the particles were calcined in air at 973 K for 6 h to remove the structure-directing agent.

#### 3.1.5. MFI-Type Zeolite

All-silica MFI-type zeolite was synthesized via a hydrothermal process [24]. Triethylamine (TEA, Tokyo Chemicals Industry) and tetraethyl orthosilicate (TEOS, Tokyo Chemicals Industry) were added to distilled water, and the mixture was stirred for 3 h at room temperature for hydrolysis. Tetrapropylammonium bromide (TPABr, Tokyo Chemicals Industry) was dissolved into the solution, and the solution was stirred for 4 h at 363 K. The molar composition of the solution was 1SiO_2_: 2TEA: 1.6TPABr: 200H_2_O. Then, the solution was poured into an autoclave, and a hydrothermal reaction was carried out at 403 K for 24 h. After cooling to room temperature, solids were recovered by filtration and washed with distilled water. The washing and filtration were repeated until the pH of the washing water became neutral, and the particles were dried in air at 383 K overnight. Finally, the particles were calcined in air at 823 K for 10 h to remove a structure-directing agent, in order to obtain all-silica MFI-type zeolite particles.

#### 3.1.6. STT-Type Zeolite

All-silica STT-type zeolite was synthesized via the same procedure as for the CHA-type zeolite [22]. The composition of a synthesis gel was 1SiO_2_: 0.5TMAAOH: 0.5HF: 10H_2_O. A hydrothermal reaction was performed at 423 K for 11 days. After solids were filtered, washed with distilled water, and dried in air at 383 K overnight, the particles were calcined in air at 823 K for 10 h for the removal of the structure-directing agent.

### 3.2. Characterization

The crystal structures of zeolites were identified via X-ray diffraction (Smart Lab., Rigaku, Tokyo, Japan), and the morphology was observed using a scanning electron microscope (TM-1000, Hitachi High Technology, Tokyo, Japan).

### 3.3. Adsorption Experiment

The amount of adsorbed CO_2_ and CH_4_ on zeolites was determined using an adsorption unit equipped with a constant volume cell (Belsorp Max, Japan BEL, Osaka, Japan) at 298 K and 343 K. Approximately 1 g of zeolite particles was added to the cell and evacuated at 673 K overnight to remove adsorbed water. The pretreated particles were used for the adsorption experiment without exposure to atmosphere. The pretreatment was carried out before each measurement. Either CO_2_ or CH_4_ were added to the cell, and the pressure was decreased because of the adsorption of these gases on the zeolite. When the pressure reached a constant level, the amount of adsorption was determined by the reduction of pressure. The difference in the amount of adsorption for each measurement was less than 1%.

### 3.4. Simulation

The adsorption isotherms of CO_2_ and CH_4_ on the all-silica BEA, CDO, CHA, DDR, MFI, and STT-type zeolites were simulated via a grand canonical Monte Carlo (GCMC) technique using a software program (Biovia, Materials Studio 2017 Sorption). For GCMC simulation, fugacity was given to the canonical ensemble, and the number and location of molecules with the lowest potential energy were calculated probabilistically. The cutoff distance of the van der Waals interaction was 1.25 nm, and the Ewald summation method was used for the integration of the coulomb interaction. The total number of Monte Carlo cycles was 10^6^, and the average of the final 10^5^ steps was used as the simulation result. The fugacity was assumed to be equal to the pressure in this study, since the difference between fugacity and pressure was less than 5% below 1 MPa.

Table 1 shows the crystal structure data of all-silica zeolites used in our calculation. The crystal structures were taken from the IZA zeolite database [21]. The bond interactions in the zeolite framework were ignored in this study.

**Figure 1:**
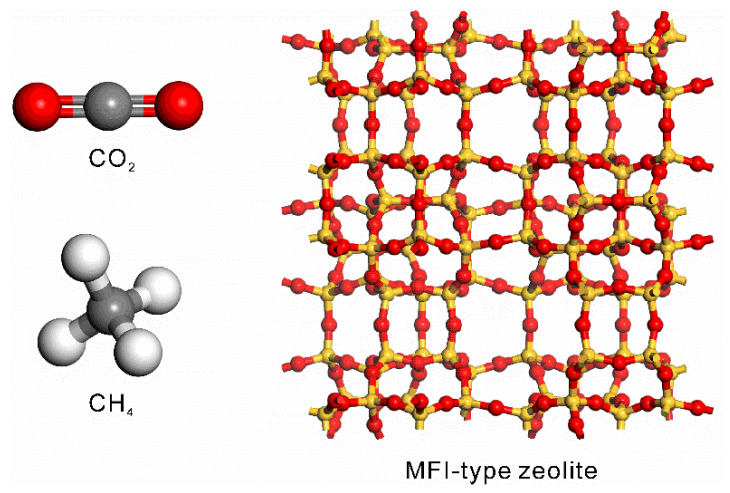
Atomistic models of CO_2_, CH_4_, and CHA-type zeolite.

Figure 1 shows the atomistic models of CO_2_, CH_4_ and MFI-type zeolite. The model of the CO_2_ molecule reported by Harris et al. [25] was used in this study. This model can describe the gas–liquid coexistence curve including the critical point region. The carbon atom was connected to two oxygen atoms by chemical bonds which are 0.1149 nm long, and bond stretching was ignored (*k*_b_ = 0). The original angle of O=C=O was 180°, and the force constant *k*_θ_ = 1236 kJ mol^−1^ rad^−2^. For CH_4_, the model reported by Siepman et al. [17] was used. The carbon atom was connected to four hydrogen atoms with the bond length of 0.11 nm, and each H-C-H angle was 109.5°. Although the bond stretching and angle bending are ignored in this model (*k*_b_ = *k*_θ_ = 0), the gas–liquid coexistence curve can be expressed. Table 2 lists the non-bonding interaction parameters for CO_2_ and CH_4_.

The self-diffusivities of CH_4_ and CO_2_ in the MFI-type zeolite channel were also simulated via a molecular dynamic technique (Biovia, Materials Studio 2017 Forcite Plus). Several molecules of CH_4_ and CO_2_ were adsorbed by GCMC, and the molecular dynamic simulation was carried with the time step of 1 fs. The total simulation time was 1 ns, and the mean square displacements of every 1 ps were plotted against the simulation time. The self-diffusivity was calculated by the Einstein equation as the slope of the mean square displacement with respect to time.

## 4. Results and Discussion

### 4.1. Characterization

**Figure 2:**
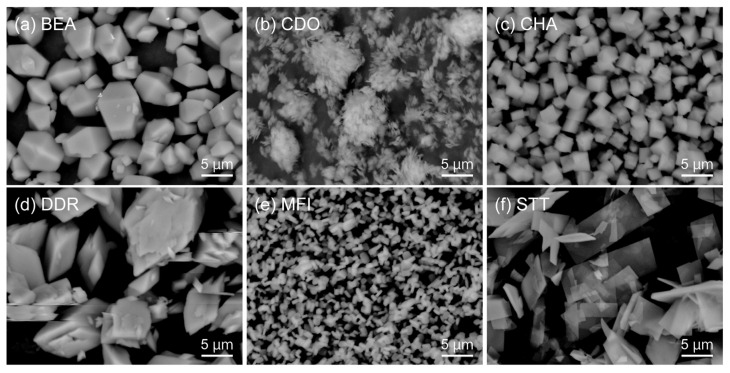
SEM images of all-silica (**a**) BEA, (**b**) CDO, (**c**) CHA, (**d**) DDR, (**e**) MFI, and (**f**) STT-type zeolite particles synthesized in this study.

**Figure 3:**
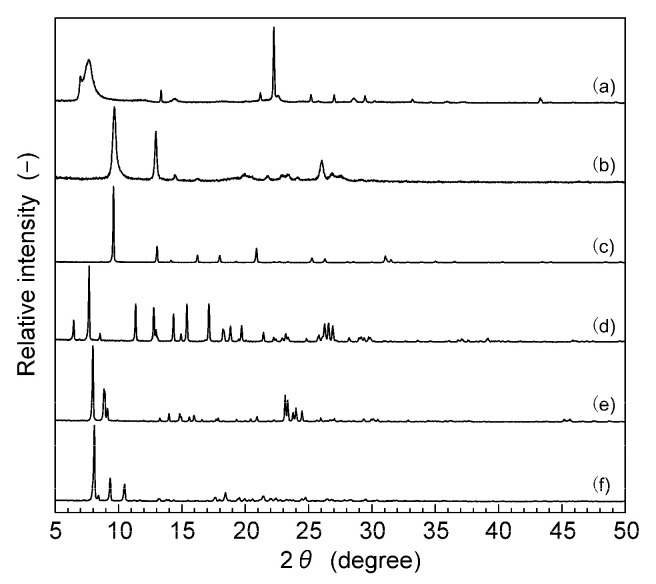
XRD patterns of all-silica (**a**) BEA, (**b**) CDO, (**c**) CHA, (**d**) DDR, (**e**) MFI, and (**f**) STT-type zeolite particles synthesized in this study.

Figure 2 shows the SEM images of the all-silica zeolite particles synthesized in this study. The shape of the BEA-type zeolite was a truncated bipyramid with the size of 2–10 μm, and the CHA-type zeolite was cubic crystals with the size of ca. 2 μm. The CDO and STT-type zeolite were plate crystallites, and the sizes were circa 2 × 2 × 0.2 μm^3^ (CDO) and 5 × 10 × 0.1 μm^3^ (STT). Figure 3 shows the XRD patterns of the zeolite particles synthesized in this study. The XRD patterns were identical to those of corresponding zeolites, and no peaks of impurities were observed. The BEA-type zeolite has two kinds of polymorphs, A and B, and the containing ratio is estimated by the peak patterns at 2*θ* = 6−8° [26]. The BEA-type zeolite particles synthesized in this study were estimated to be A/B = 1/1. These results suggest that the zeolite crystallites with no impurities can be synthesized.

### 4.2. Adsorption Isotherm

**Figure 4:**
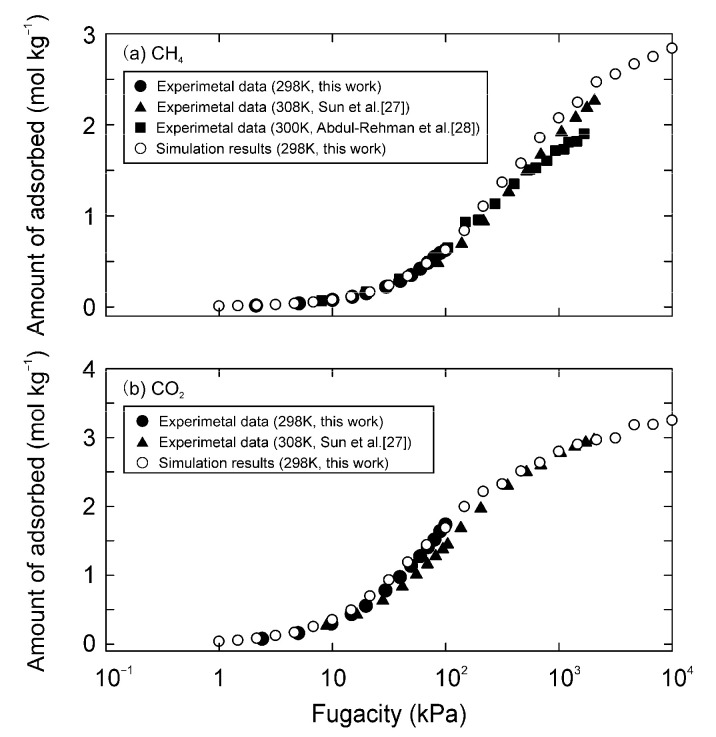
Adsorption isotherms of (**a**) CH_4_ and (**b**) CO_2_ on all-silica MFI-type zeolite around 300 K.

**Figure 5:**
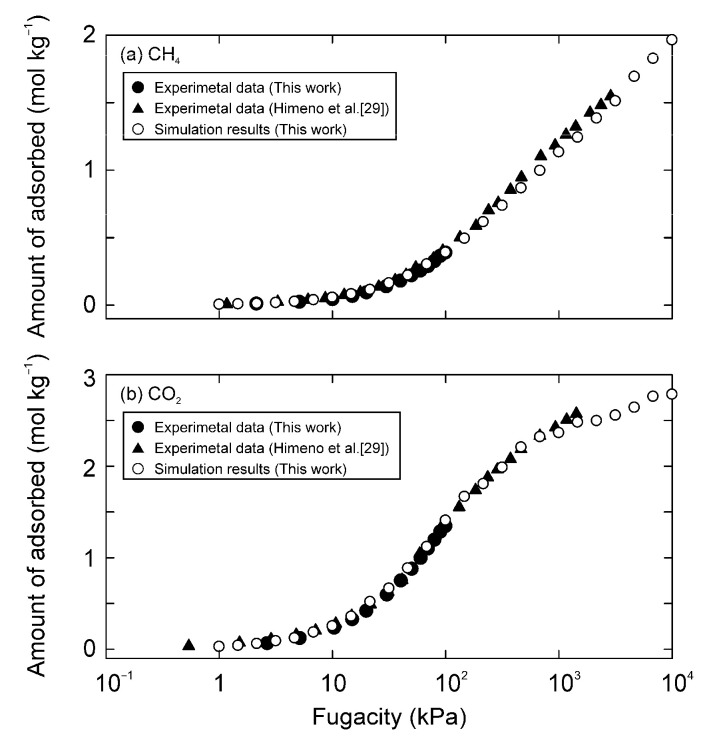
Adsorption isotherms of (**a**) CH_4_ and (**b**) CO_2_ on all-silica DDR-type zeolite at 298 K.

Figure 4 and Figure 5 compare the simulated adsorption isotherms of CO_2_ and CH_4_ on the MFI and DDR-type zeolites to experimental results, respectively. Sun et al. [27] and Abdul-Rehman et al. [28] reported the adsorption isotherms of CO_2_ and CH_4_ on MFI-type zeolites at high pressures, and those for the DDR-type zeolite were reported by Himeno et al. [29]. Our experimental results agreed well with these reported data. The parameters were then optimized, so that the simulation results matched the experimental data. As a result, the parameters listed in Table 3 were obtained. The simulation results were more consistent with the experimental data by giving the van der Waals parameters to only the Si atom of zeolites, as compared to cases where the parameters were given to both O and Si atoms. The distance between the Si atoms at zero interaction potential σ was the same as the van der Waals diameter of Si (0.42 nm [30]), and the potential depth of ε was close to the interaction potential calculated by the Kirkwood–Muller equation (1.0 kJ mol^−1^) [31]. Furthermore, assuming that the bond length of Si-O is 0.15 nm, the partial charge of the Si atom could be calculated to be 1.02 e from the dipole moment of the Si-O bond (1.23 × 10^−29^ C m [32]). These suggest that our parameters are theoretically reasonable.

**Figure 6:**
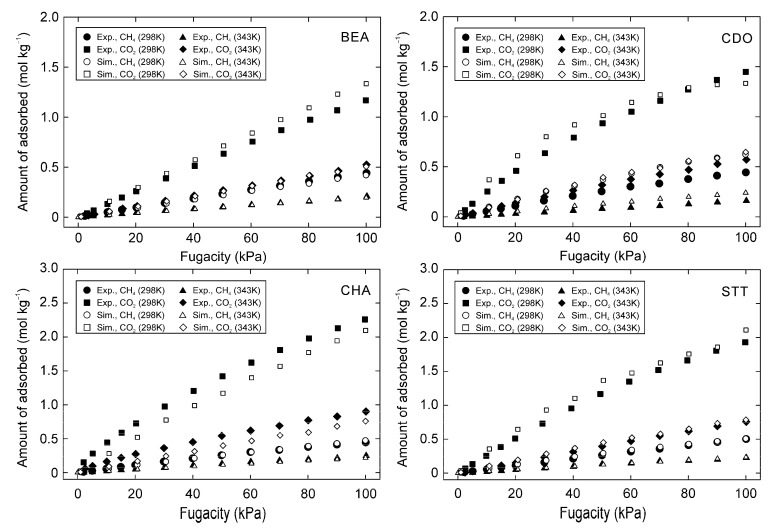
Adsorption isotherms of CH_4_ and CO_2_ on all-silica (**top left**) BEA, (**top right**) CDO, (**bottom left**) CHA, and (**bottom right**) STT-type zeolite at 298 K and 343 K.

**Figure 7:**
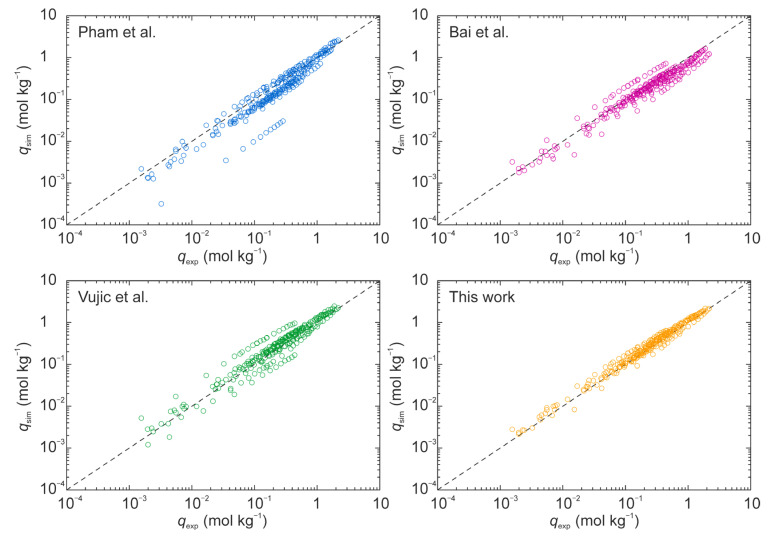
Comparison of simulated amount of adsorbed CO_2_ and CH_4,_ using several parameters of the experimental data.

Figure 6 compares the simulated adsorption isotherms of CH_4_ and CO_2_ on BEA, CDO, CHA, and STT-type zeolites to the experimental data. The simulated values were almost the same as the experimental results, and the differences were below 0.09 mol kg^−1^ for CH_4_, and 0.30 mol kg^−1^ for CO_2_. The same amounts of adsorbed CO_2_ and CH_4_ at different temperatures gave the same heats of adsorption between experiments and simulation. As described below, the isotherms on the BEA-type zeolite had the largest differences in previous studies. The heat of adsorption on the BEA-type zeolite was 18.0 kJ mol^−1^ for CO_2_ and 15.3 kJ mol^−1^ for CH_4_ experimentally, and the simulated values were 18.5 and 14.6 kJ mol^−1^ for CO_2_ and CH4, respectively. Pham et al. [11], Bai et al. [13], and Vujic et al. [14] reported the non-bonding parameters for zeolites, as shown in Table 4, and they also simulated the adsorption isotherms on several kinds of zeolites. The adsorption isotherms of CO_2_ and CH_4_ on the BEA, CDO, CHA, DDR, MFI, and STT-type zeolites were also simulated using their parameters in this study. Their parameters could thus predict the adsorption isotherms on some zeolites. However, the accuracies for the adsorption on the BEA and CDO-type zeolites were significantly low. Figure 7 compares the simulated adsorption amount using their parameters for the experimental data, and Table 4 summarizes the average difference between the experimental and simulated values and its standard deviation. The average of difference and standard derivation were calculated as follows:

(10) $$\mathrm{Average}\;\mathrm{of}\;\mathrm{difference}\;(\%)=\frac{100}{N}\sum_{}^{N}\frac{\left|q_{\exp}-q_{\mathrm{sim}}\right|}{q_{\exp}},$$

(11) $$\mathrm{Standard}\;\mathrm{derivation}\;(\%)=\frac{100}{N}\sum_{}^{N}|\frac{\left|q_{\exp}-q_{\mathrm{sim}}\right|}{q_{\exp}}-\frac{1}{N}\sum_{}^{N}\frac{\left|q_{\exp}-q_{\mathrm{sim}}\right|}{q_{\exp}}|,$$

where *q*_exp_ and *q*_sim_ are the amount of adsorbed obtained by the experiment and simulation, respectively. The average of the difference and standard deviation of our parameters were 0.06 mol kg^−1^ and 0.06 mol kg^−1^, respectively. These were lower than those for the other parameters. Therefore, we conclude that our parameters can predict the amounts of adsorbed CO_2_ and CH_4_ on all-silica zeolites with high accuracy.

### 4.3. Self-Diffusivity Adsorption Isotherm

**Figure 8:**
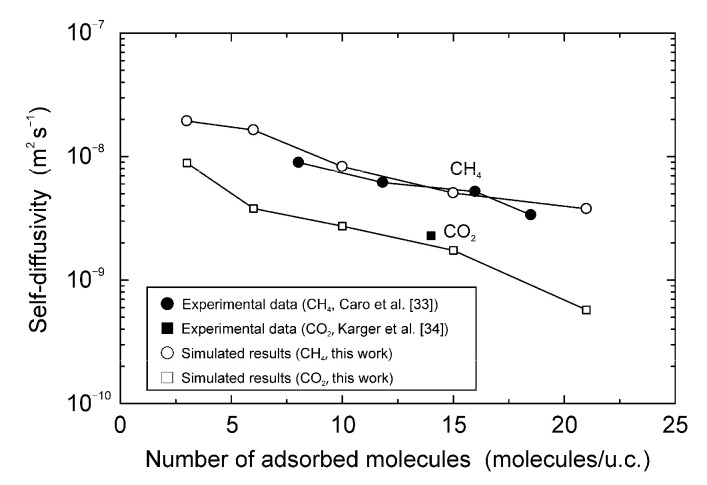
Influences of the number of adsorbed molecules on the self-diffusivities of CH_4_ and CO_2_ at 298 K.

Next, the self-diffusivities were simulated via molecular dynamics to confirm the effectiveness of our parameters. The MFI-type zeolite was the selected zeolite, since many experimental data of self-diffusivities are reported. Figure 8 shows the influences of the amount of adsorbed CO_2_ and CH_4_ on the self-diffusivities of CO_2_ and CH_4_ in the MFI-type zeolite at 298 K. When three CH_4_ molecules were adsorbed to the unit cell (0.52 mol kg^−1^), the diffusivity was estimated to be 1.9 × 10^−8^ m^2^ s^−1^. The diffusivity decreased with an increase in the number of adsorbed molecules, and it was 3.8 × 10^−9^ m^2^ s^−1^ per twenty-one CH_4_ molecules adsorbed (3.64 mol kg^−1^). The self-diffusivity of CO_2_ showed the same trend. Caro et al. [33] and Karger et al. [34] used the PFR-NMR method to measure the self-diffusivities of CH_4_ and CO_2_ in the all-silica, MFI-type zeolite. Their data are also plotted in Figure 8. Our simulation results were in good agreement with their experimental results.

**Figure 9:**
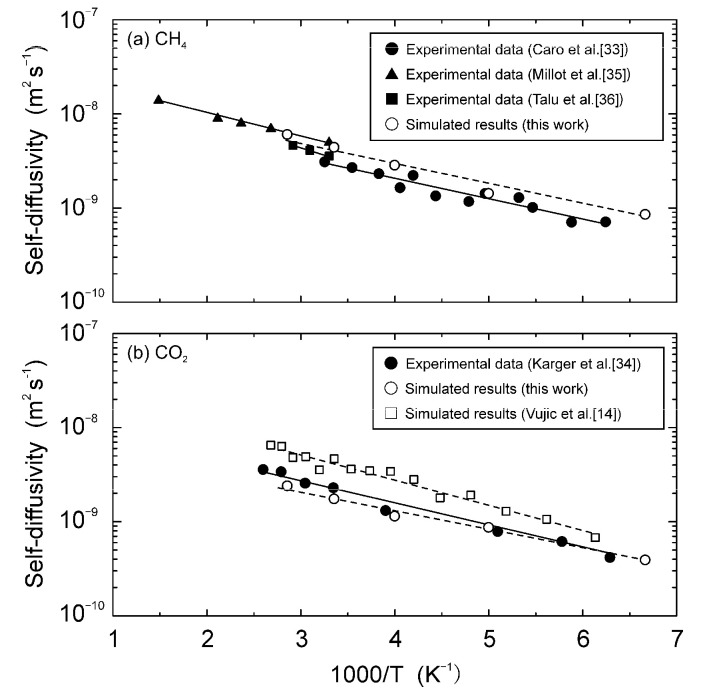
Effects of temperatures on the self-diffusivities of (**a**) CH_4_ and (**b**) CO_2_.

Finally, the effect of temperatures on the self-diffusivities of CH_4_ and CO_2_ in MFI-type zeolite was simulated. Figure 9 shows the temperature dependencies of the self-diffusivities of CO_2_ and CH_4_ in the MFI-type zeolite. For the comparison with the PFG-NMR experimental data of Caro et al. [33] and Karger et al. [34], nineteen CH_4_ or fifteen CO_2_ molecules were adsorbed, and the molecular dynamic simulations were carried out. These number of molecules correspond to the amount of adsorbed 3.29 or 2.60 mol kg^−1^, respectively. Millot et al. [35] and Talu et al. [36] determined the diffusivities from the permeability of CH_4_ through a polycrystalline membrane and single crystal membrane, respectively. The self-diffusivities of CH_4_ simulated using our parameters were similar to the experimental results, as shown in Figure 9a. Further, Vujic et al. [14] simulated the CO_2_ diffusivity in the MFI-type zeolite by their non-binding parameters, as listed in Table 4. However, their simulation results are 2–3 times higher than the experimental results of Karger [34], as shown in Figure 9b. By contrast, the CO_2_ diffusivities using our parameters were close to the experimental results. This means that our parameters can be used for the prediction of the self-diffusivities in MFI-type zeolite with a higher accuracy than previously reported parameters.

## 5. Conclusions

In this study, all-silica BEA, CDO, CHA, DDR, MFI, and STT-type zeolite particles were synthesized, and the amount of adsorbed CO_2_ and CH_4_ was determined at 298 K and 343 K. When the non-bonding parameters (*σ*_Si_ = 0.421 nm, *ε*_Si_ = 0.954 kJ mol^−1^, and *q*_Si_ = 1.10) were used, the simulated adsorption isotherms showed better agreement with the experimental results than those calculated using previously reported parameters. Those parameters also provided self-diffusivities close to experimental data, compared to the other parameters. These results suggest that our derived parameters are useful for selecting zeolite structure as the membrane material.

## Figures and Tables

**Table 1:** Crystal structures of all-silica zeolites.

Zeolite	Space Group	*a* (nm)	*b* (nm)	*c* (nm)	*α* (°)	*β* (°)	*γ* (°)	Composition
BEA	P4_1_22 (No.91)	1.266	1.266	2.641	90.00	90.00	90.00	Si_64_O_128_
CDO	Pnma (No.62)	1.836	1.378	7.367	90.00	90.00	90.00	Si_36_O_72_
CHA	R3m (No.166)	0.942	0.942	0.942	94.07	94.07	94.07	Si_12_O_24_
DDR	R3m (No.166)	1.386	1.386	4.089	90.00	90.00	120.00	Si_120_O_240_
MFI	Pnma (No.62)	2.002	1.990	1.338	90.00	90.00	90.00	Si_96_O_192_
STT	P2_1_/N (No.14)	1.309	2.167	1.373	90.00	102.58	90.00	Si_64_O_128_

**Table 2:** Non-bonding interaction parameters of CH_4_ and CO_2_.

Molecule	Element	*σ* (nm)	*ε*/*k* (K)	*q* (e)	Ref.
CH_4_	C	0.3730	148.0	0	[17]
	H	---	---	0	
CO_2_	C	0.2757	28.1	0.6512	[25]
	O	0.3033	80.5	−0.3256	

**Table 3:** Non-bonding interaction parameters of all-silica zeolites.

Elements	*σ* (nm)	*ε* (kJ mol^−1^)	*q* (e)
Si	0.421	0.954	1.10
O	---	---	−0.55

**Table 4:** Non-bonding interaction parameters of all-silica zeolites, averages of differences between the amounts of adsorbed obtained by the experiment and simulation, and their standard deviations.

Element	Parameter	Bai et al. [11]	Pham et al. [13]	Vujic et al. [14]	This Work
Si	*σ* (nm)	0.231	---	0.297	0.421
	*ε* (kJ mol^−1^)	0.185	---	0.266	0.954
	*q* (e)	1.5	2.0	1.413	1.10
O	*σ* (nm)	0.3304	0.2806	0.3011	---
	*ε* (kJ mol^−1^)	0.442	0.744	0.432	---
	*q* (e)	−0.75	−1.0	−0.7065	−0.55
Average of difference (mol kg^−1^)		0.09	0.13	0.09	0.06
Standard deviation (mol kg^−1^)		0.09	0.18	0.10	0.06

## Data Availability

Not applicable.

## Nomenclatures

*E*	potential energy	(kJ mol^−1^)
*k*_b_	force constant for bond stretching	(kJ mo^−1^ m^−2^)
*k_θ_*	force constant for angle bending	(kJ mo^−1^ rad^−2^)
*q*	partial atomic charge	(e)
*r*	distance	(m)
Greeks		
*ε*	depth of potential	(kJ mol^−1^)
*ε*_0_	vacuum permittivity	(=8.85 × 10^−12^ F m^−1^)
*θ*	bonding angle	(rad)
*σ*	distance at zero-potential energy	(m)
Subscripts		
0	original position	
*i*	component *i*	
*ij*	pair of atoms *i* and *j*	

## References

[1] Sano T., Kiyozumi Y., Kawamura M., Mizukami F., Takaya H., Mouri T., Inaoka W., Toida Y., Watanabe M., Toyoda K. (1991). Preparation and characterization of ZSM-5 zeolite films. Zeolites.

[2] Geus E.R., den Exter M.J., van Bekkum H. (1992). Synthesis and characterization of zeolite (MFI) membranes on porous ceramic supports. J. Chem. Soc. Faraday Trans..

[3] Hasegawa Y., Matsuura W., Abe C., Ikeda A. (2021). Influence of organic solvent species on dehydration behaviors of NaA-type zeolite membrane. Membranes.

[4] Inami H., Abe C., Hasegawa Y. (2021). Development of ammonia selective permeable zeolite membrane for sensor in sewer system. Membranes.

[5] Imasaka S., Itakura M., Yano K., Fujita S., Okada M., Hasegawa Y., Abe C., Araki S., Yamamoto H. (2018). Rapid preparation of high-silica CHA-type zeolite membranes and their separation properties. Sep. Purif. Technol..

[6] Crreon M.A., Li S., Falconer J.L., Noble R.D. (2008). Alumina-supported SAPO-34 membranes for CO_2_/CH_4_ separation. J. Am. Chem. Soc..

[7] Van den Bergh J., Zhu W., Gascon J., Moulijn J.A., Kapteijn F. (2008). Separation and permeation characteristics of a DD3R zeolite membrane. J. Membr. Sci..

[8] Suzuki S., Takaba H., Yamaguchi T., Nakao S. (2000). Estimation of gas permeability of a zeolite membrane, based on a molecular simulation technique and permeation model. J. Phys. Chem. B.

[9] Talu O., Myers A.L. (2001). Reference potentials for adsorption of helium, argon, methane, and krypton in high-silica zeolites. Colloids Surf. A.

[10] Makrodimitris K., Papadopoulos G.K., Theodorou D.N. (2001). Prediction and permeation properties of CO_2_ and N_2_ through silicalite via molecular simulations. J. Phys. Chem. B.

[11] Bai P., Tsapatsis M., Siepmann J.I. (2013). TraPPE-zeo: Transferable potentials for phase equilibria force field for all-silica zeolites. J. Phys. Chem. C.

[12] Rahmati M., Modarress H. (2013). Selectivity of new siliceous zeolites for separation of methane and carbon dioxide by Monte Carlo simulation. Micropor. Mesopor. Mater..

[13] Pham T.D., Xiong R., Dandler S.I., Lobo R.F. (2014). Experimental and computational studies on the adsorption of CO_2_ and N_2_ on pure silica zeolites. Micropor. Mesopor. Mater..

[14] Vujic B., Lyubartsev A.P. (2016). Transferable forcefield for modelling of CO_2_, N_2_, O_2_, and Ar in all-silica and Na^+^ exchanged zeolites. Model. Simul. Mater. Sci. Eng..

[15] Sun H. (1998). COMPASS: An ab initio force-field optimized for condensed phase application-overview with details on alkane and benzene compounds. J. Phys. Chem. B.

[16] Yang J., Ren Y., Tian A., Sun H. (2000). COMPASS force field for 14 inorganic molecules, He, N_2_, Ar, Kr, Xe, H_2_, O_2_, N_2_, NO, CO, CO_2_, NO_2_, CS_2_, and SO_2_ in liquid phase. J. Phys. Chem. B.

[17] Martin M.G., Siepmann J.I. (1998). Transferable potentials for phase equilibria. 1. United-atom description of n-alkanes. J. Phys. Chem. B.

[18] Martin M.G., Siepmann J.I. (1999). Novel configurational Monte Carlo method for branched molecules. Transferable potentials for phase equilibria. 2. United-atom description of branched alkanes. J. Phys. Chem. B.

[19] Wick C.D., Martin M.G., Siepmann J.I. (2000). Transferable potentials for phase equilibria. 4. United-atom description of linear and branched alkanes and alkylbenzenes. J. Phys. Chem. B.

[20] Potoff J.J., Siepmann J.I. (2001). Vapor-liquid equilibria of mixtures containing alkanes, carbon dioxide, and nitrogen. AIChE J..

[21] Camblor M.A., Diaz-Cabanas M.J. (1999). A Synthesis, MAS NMR, Synchrotron X-ray PowderDiffraction, and Computational Study of Zeolite SSZ-23. Chem. Mater..

[22] Ikeda T., Akiyama Y., Oumi Y., Kawai A., Mizukami F. (2004). The toporactic conversion of a novel layered silicate into a new framework zeolites. Angew. Chem. Int. Ed..

[23] den Exter M.J., Jansen J.C., van Bekkum H. (1994). Separation of Permanent Gases on the All-Silica 8-Ring Clathrasil DD3R. Stud. Surf. Sci. Catal..

[24] Ban T., Takahashi Y., Ohya Y., Ozeki Y., Yoshikawa M. JP2003-238147.

[25] Harris J.G., Yung K.H. (1995). Carbon dioxide’s liquid-vapor coexistence curve and critical properties as predicted by a simple molecular model. J. Phys. Chem..

[26] Treacy M.M.J., Higgins J.B. (2007). Collection of Simulated XRD Powder Patterns for Zeolites.

[27] Sun M.S., Shah D.B., Xu H.H., Talu O. (1998). Adsorption Equilibria of C_1_ to C_4_ Alkanes, CO_2_, and SF_6_ on Silicalite. J. Phys. Chem. B.

[28] Abdul-Rehman H.B., Hasanain M.A., Loughlin K.F. (1990). Quaternary, Ternary, Binary, and Pure Component Sorption on Zeolites. 1. Light Alkanes on Linde S-115 Silicalite at Moderate to High Pressures. Ind. Eng. Chem. Res..

[29] Himeno S., Tomita T., Suzuki K., Yoshida S. (2007). Characterization and selectivity for methane and carbon dioxide adsorption on the all-silica DD3R zeolite. Micropor. Mesopor. Mater..

[30] Batsanov S.S. (2001). Van der Waals Radii of Elements. Inorg. Mater..

[31] Rege S.U., Yang R.T. (2000). Corrected Horvath-Kawazoe equations for pore-size distribution. AIChE J..

[32] Raymonda J.W., Muenter J.S., Klemperer W.A. (1970). Electric Dipole Moment of SiO and GeO. J. Chem. Phys..

[33] Caro J., Bulow M., Schirmer W. (1985). Microdynamics of methane, ethane and propane in ZSM-5 type zeolites. J. Chem. Soc. Faraday Trans..

[34] Karger J., Pfeifer H., Stallmach F. (1993). ^129^Xe and ^13^C PFG n.m.r. study of the intracrystalline self-diffusion of Xe, CO_2_ and CO. Zeolites.

[35] Millot B., Methivier A., Jobic H., Moueddeb H., Dalmon J.A. (2000). Permeation of linear and branched alkanes in ZSM-5 supported membranes. Micropor. Mesopor. Mater..

[36] Talu O., Sun M.S., Shah S.B. (1998). Diffusivities of n-alkanes in silicalite by steady-state single-crystal membrane technique. AIChE J..

